# Total Neoadjuvant Chemotherapy Followed by Laparoscopic Radical Gastrectomy Versus Neoadjuvant Chemotherapy for Clinical Stage T4a-bN + M0 Proximal Gastric Cancer: A Single-Center Retrospective Data Analysis

**DOI:** 10.1155/grp/4020664

**Published:** 2025-12-03

**Authors:** ChangRong Que, ShuangMing Lin, Wen Ye, RunSheng Lai, Run Xie, DongBo Xu

**Affiliations:** Department of Gastrointestinal Surgery, Longyan First Hospital Affiliated to Fujian Medical University, Longyan, Fujian Province, China

**Keywords:** gastric cancer, surgery, total neoadjuvant chemotherapy

## Abstract

**Background:**

While preoperative neoadjuvant chemotherapy (NT) followed by surgery has gained acceptance in the management of locally advanced gastric cancer (LAGC), there remains a paucity of studies examining the efficacy of total neoadjuvant chemotherapy (TNT) for LAGC. This study was aimed at addressing this gap by comparing the outcomes of patients with clinical stage T4a-bN + M0 proximal gastric cancer treated with TNT and NT. The investigation sought to provide valuable insights into the effectiveness of the TNT regimen in this specific clinical context.

**Methods:**

Retrospective analysis was conducted on the clinical data of patients diagnosed with proximal LAGC who underwent perioperative docetaxel, oxaliplatin, and fluorouracil (FLOT) chemotherapy followed by laparoscopic radical gastrectomy at Longyan First Hospital affiliated with Fujian Medical University. The study, spanning from January 2017 to December 2019, included 26 patients in the TNT group and 32 patients in the NT group. Comparative assessments were made regarding the outcomes of chemotherapy and surgery, as well as the 3-year disease-free survival (DFS) and overall survival (OS) between the two groups.

**Results:**

The TNT group demonstrated superiority over the NT group in terms of operation time and intraoperative blood loss. While no significant difference was observed in total postoperative complications between the two groups, the TNT group exhibited a more pronounced downstaging in T stage and a higher rate of pathological complete regression. The 3-year OS rate was notably higher in the TNT group at 61.5%, compared to 46.9% in the NT group. Similarly, the 3-year DFS rate favored the TNT group at 53.8%, surpassing the rate of 34.4% in the NT group.

**Conclusions:**

The TNT approach for LAGC had the potential to enhance tumor regression and increase the completion rate of chemotherapy. This strategy demonstrated a positive trend in long-term outcomes and introduced a novel treatment model.

## 1. Introduction

As widely recognized, approximately 42% of global gastric cancer (GC) cases occur in China [1], ranking third among prevalent malignant tumors [2]. Notably, about 70.8% of these cases manifest as locally advanced gastric cancer (LAGC). Over the past decade, the therapeutic landscape for LAGC has evolved from exclusive surgical interventions to a multidisciplinary treatment approach anchored in surgery. Consequently, preoperative neoadjuvant chemotherapy (NT) followed by surgery has gained gradual acceptance [3–5]. Despite this shift, there remains a lack of consensus regarding the optimal regimen and duration for preoperative NT. Previous studies have typically recommended two to four cycles of chemotherapy. However, some patients continue to exhibit substantial tumor sizes even after completing 4 cycles of chemotherapy. Consequently, investigating the potential benefits of adopting an extended preoperative chemotherapy cycle for achieving superior tumor regression represents an imperative area of exploration. Complicating matters, many GC patients encounter challenges in completing the postoperative chemotherapy regimen due to surgical trauma and physiological changes. In light of these considerations, we have devised a novel preoperative treatment strategy involving total neoadjuvant chemotherapy (TNT) followed by surgery, aimed at optimizing outcomes. Notably, there is limited literature on such an approach within the domain of GC research.

## 2. Methods

### 2.1. Patient Selection

We retrospectively collected clinical data from patients diagnosed with proximal LAGC who underwent perioperative treatment with docetaxel, leucovorin, oxaliplatin, and fluorouracil (FLOT) followed by laparoscopic radical gastrectomy at Longyan First Hospital affiliated with Fujian Medical University between January 2017 and December 2019.

The FLOT treatment regimen included intravenous administration of docetaxel (50 mg/m^2^) on Day 1, oxaliplatin (85 mg/m^2^) on Day 1, leucovorin (200 mg/m^2^) on Day 1, and fluorouracil (2600 mg/m^2^) as a 48-h infusion on Day 1. This treatment was administered every 2 weeks. The inclusion criteria were defined as follows: age between 20 and 70 years, histologically proven moderately differentiated adenocarcinoma of the stomach, proximal third location of gastric or gastroesophageal junction, clinical TNM staging T4a-bN + M0 before initial treatment, resectable or potentially resectable tumor, no previous radiotherapy or chemotherapy, partial tumor remission after preoperative four cycles of FLOT chemotherapy, Eastern Cooperative Oncology Group performance status 0–1, and no myelosuppression before initial treatment. The exclusion criteria were defined as follows: history of abdominal surgery, emergency surgery due to tumor perforation or bleeding, allergies to drugs used in chemotherapy, major organ dysfunction or uncontrolled basic diseases, combined with other malignant tumors. Tumor staging was based on the seventh edition of the pathological (pTNM) classification of the American Joint Committee on Cancer (AJCC). Following the criteria, 58 patients were screened. The 58 patients were comprehensively informed about the subsequent treatment options, including either immediate surgery or continuation of chemotherapy. The potential benefits and risks associated with each approach were thoroughly explained, and the final decision was made based on the patients' informed preferences. Among them, 26 patients who continued to receive four cycles of FLOT chemotherapy followed by surgery were categorized into the TNT group, while 32 patients who received surgery followed by four cycles of FLOT chemotherapy were categorized into the NT group. All patients included in the study provided written informed consent. The study protocol was conducted in accordance with the ethical principles outlined in the Declaration of Helsinki and was approved by the Ethics Committee of Longyan First Hospital affiliated with Fujian Medical University.

### 2.2. Procedures

Prior to the initiation of treatment, all patients underwent a comprehensive set of baseline examinations, including blood routine examination, biochemistry, tumor markers, electrocardiogram, and assessment of cardiopulmonary function. The confirmation of tumor type involved obtaining biopsy tissue through electronic gastroscopy. Tumor staging was evaluated through abdominal and chest enhanced computed tomography (CT) examinations. In cases where additional precision was needed, liver magnetic resonance imaging (MRI) or positron emission tomography-computed tomography (PET-CT) was employed. The determination of tumor staging was a collaborative effort facilitated by an experienced multidisciplinary tumor team. This team comprised experts from various departments, including imaging, gastrointestinal surgery, pathology, hepatobiliary surgery, thoracic surgery, and oncology. Patients, who had previously undergone four cycles of the FLOT regimen with confirmed partial remission through enhanced CT evaluation, were subsequently categorized into two groups: TNT group and NT group. For the NT group, laparoscopic total gastrectomy with D2 lymphadenectomy was the initial surgical procedure. In cases where the tumor was located at the gastroesophageal junction, partial resection of the esophagus through the esophageal hiatus was performed. Postoperative chemotherapy, starting within a month after surgery, constituted the first cycle. The TNT group continued to receive four cycles of FLOT chemotherapy and underwent the same surgical treatment as the NT group 3weeks after completing all chemotherapy cycles. Standard prechemotherapy measures were implemented for all patients, involving the administration of dexamethasone to prevent allergies and the prophylactic use of two antiemetics to minimize gastrointestinal reactions. Routine enteral or intravenous nutrition support was provided to manage adverse reactions associated with chemotherapy. In cases of leukopenia, granulocyte-stimulating factor was administered to stimulate leukocyte growth. Additionally, recombinant human platelet-stimulating factor was utilized to support platelet growth. Chemotherapy drug doses were reduced to 75% for patients experiencing Grade 3 adverse reactions. A further reduction to 50% was applied in cases where toxicities recurred post the initial dose adjustment. Criteria for stopping treatment were unacceptable toxicity, disease progression, request of patient, or decision of investigator that stopping treatment was in the best interest of the patient. Experienced gastrointestinal surgeons within the same group conducted all surgical operations. Surgical specimens were promptly fixed in formalin solution within 30 min post-isolation. Examination involved measuring macroscopically identifiable residual tumors or scarring indicating the previous tumor bed. Specimens underwent staining (hematoxylin and eosin, Elastica van Gieson, and periodic acid-Schiff) to detect tumor desmoplasia, scarring, and signet-ring cells. In cases with no tumor cells found, the entire suspected original tumor bed was tested for confirmation. An experienced pathologist confirmed tumor regression grade (TRG) and pathological staging through histopathological sections.

Chemotherapy-related adverse events were classified per the World Health Organization standard for general toxic drugs. Postoperative complications were classified using the Clavien–Dindo classification. TRGs followed the NCCN TRG grading standard.

## 3. Follow-Up

The follow-up time was 36 months. Follow-up methods included telephone calls, outpatient records, and hospital examination. Follow-up was performed every 3 months until 3 years after diagnosis, and there was no loss of patients.

## 4. Statistical Analysis

All data were statistically analyzed using SPSS 27.0 software (SPSS Inc., Chicago, IL, United States). Categorical variables were analyzed using the Chi-squared or Fisher's exact test, whereas continuous variables were analyzed using the unpaired Student's *t*-test. Cumulative survival rates were estimated using the Kaplan–Meier method and compared with the log-rank test. Two-sided *p* values less than 0.05 were considered to be significant.

## 5. Results

The study included 26 patients in the TNT group and 32 patients in the NT group. There was no statistically significant difference between the two groups regarding gender, age, ECOG score, NRS2002 score, tumor location, and clinical stage (Table 1). It is noteworthy that the baseline data for both groups were collected prior to the initial treatment rather than after four cycles of chemotherapy with the FOLT regimen.

All enrolled patients underwent laparoscopic total gastrectomy with D2 lymphadenectomy and partial esophagectomy if the tumor was located at the gastroesophageal junction. Within the TNT group, one case involved combined splenectomy. In the NT group, three cases underwent combined splenectomy and partial pancreatectomy. No serious intraoperative complications occurred. Despite no significant difference in the number of lymph node resections between the two groups, the TNT group exhibited superiority over the NT group in terms of operation time and intraoperative blood loss (Table 2).

Rigorous examination of all surgical specimens was conducted by specially trained pathologists. The results for pathological regression were shown in Table 2.A significantly higher proportion of patients in the TNT group (five out of 26 patients) achieved pathological complete regression (defined as tumor regression grade 0 (TRG0)) compared to the NT group (two out of 32 patients). The TNT group exhibited an 88% pathological response rate (23 out of 26 patients), while the NT group had a response rate of 56% (18 out of 32 patients). The TNT group demonstrated a significant advantage in pathological tumor regression compared to the NT group.

In the TNT group, 25 cases were resected with R0, and one case with R1 due to a small amount of residual tumor cells at the upper margin. In the NT group, 30 cases were resected with R0. Two patients did not obtain R0 resection due to a positive upper margin or invasion of the celiac trunk artery. In terms of tumor stage, the TNT group exhibited more pronounced downstaging in T stage. Patients with pathological stage T0–T2 in the TNT group accounted for 76.9%,which was higher than 37.5% in the NT group.

No significant difference in total postoperative complications was observed between the TNT and NT groups (Table 3). Some cases experienced multiple complications simultaneously. Complication severity ranged from Clavien Dindo I to III. All complications, irrespective of severity, were effectively addressed through medical intervention or drainage via percutaneous puncture after local anesthesia. Notably, no complications classified as Clavien Dindo IV or V were identified in either the TNT or NT groups.

In the TNT group, all patients received chemotherapy with the FLOT regimen for eight cycles before the operation, despite some undergoing a reduction in dose or delay in administration time. In contrast, despite comprehensive measures to mitigate adverse reactions, 16 patients in the NT group failed to complete the scheduled four cycles of postoperative chemotherapy. The primary chemotherapy-related adverse events observed in enrolled patients were vomiting, myelosuppression, and liver dysfunction. Importantly, there were no instances of chemotherapy-related deaths during the treatment period. There was no significant difference in the incidence of chemotherapy-related adverse events between the TNT and NT groups (Table 4).

**Figure 1:**
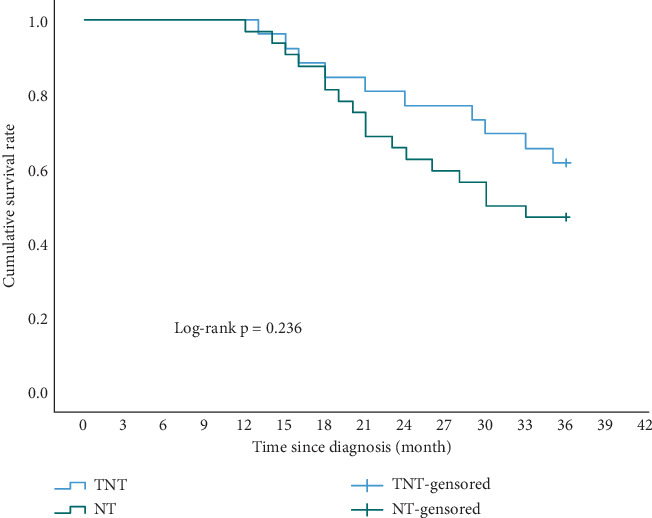
Comparison of the 3-year overall survival rates between the TNT and NT groups.

**Figure 2:**
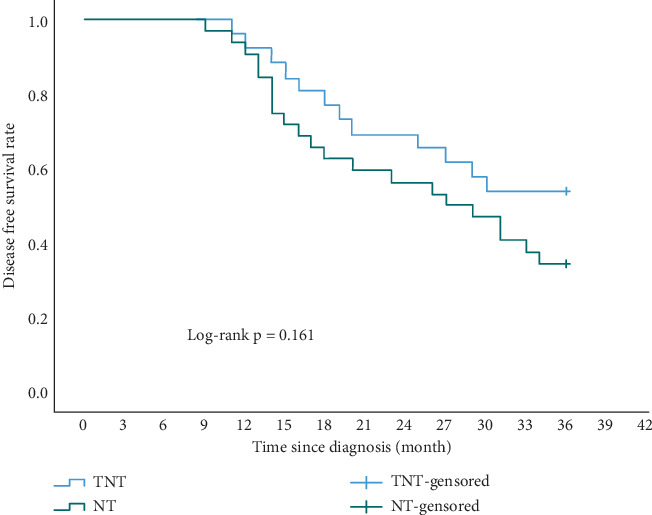
Comparison of the 3-year disease-free survival rates between the TNT and NT groups.

Enrolled patients underwent a continuous 3-year follow-up, and there were no lost cases in either group. In the TNT group, 12 patients experienced disease progression. Sites of recurrence or metastasis included peritoneum (four cases), retroperitoneal lymph nodes (three cases), liver (three cases), lung (one case), and esophagojejunal anastomosis (one case). Then, 10 patients in the TNT group died due to tumor recurrence. In the NT group, 21 patients experienced disease progression. Sites of recurrence or metastasis included peritoneum (six cases), retroperitoneal lymph nodes (five cases), liver (four cases), lung (one case), bone (one case), esophagojejunal anastomosis (three cases), and left supraclavicular lymph nodes (one case). Then, 16 patients in the NT group died due to tumor recurrence, and one patient died of myocardial infarction. The 3-year overall survival (OS) rate was 61.5% in the TNT group and 46.9% in the NT group (*p* = 0.236) (Figure 1). The 3-year disease-free survival (DFS) rate was 53.8% in the TNT group and 34.4% in the NT group (*p* = 0.161) (Figure 2). While not statistically significant, the TNT group exhibited clinically beneficial trends in long-term tumor outcomes.

## 6. Discussion

In the realm of NT for GC, experts specializing in gastric tumors have undertaken numerous initiatives. The European MAGIC study stood out, affirming that preoperative neoadjuvant treatment can enhance OS and DFS in patients with LAGC for the first time, thereby proposing a novel treatment paradigm [6]. Subsequent clinical research, such as the FLOT4-AIO study, demonstrated that a triple chemotherapy regimen comprising docetaxel, oxaliplatin, and fluorouracil can further improve the R0 resection rate and pathological response rate. Notably, the 5-year OS rate reached 45%, with a DFS of 41% [7, 8]. In the Asian context, the RESOLVE study [9] in China adopted the SOX scheme, and the PRODIGY study [10] in South Korea employed the DOS scheme, both yielding positive efficacy outcomes. In comparison, the FLOT4-AIO study enrolled a higher proportion of patients with tumors located at the gastroesophageal junction. Observations during GC treatment revealed that even after four cycles of NT, tumors remained substantial in size for patients with high local tumor loads before initial treatment. This raised concerns about increased surgical difficulty and the likelihood of tumor residue. Consequently, a strategy was implemented to encourage patients to complete all chemotherapy cycles before surgery, aiming for more significant tumor regression. Despite the theoretical improvement in LAGC outcomes with neoadjuvant therapy, challenges arise with prolonged chemotherapy cycles, particularly with the FLOT triplet regimen. The FLOT4-AIO study reported that only 46% of patients completed preoperative and postoperative chemotherapy as planned, potentially impacting long-term tumor outcomes. This phenomenon was hypothesized to be linked to surgical trauma, postoperative complications, and substantial changes in nutrient intake after total gastrectomy. Efforts should be made to enhance chemotherapy tolerance. Existing evidence suggests that nutritional disorders play a crucial role in reducing patients' tolerance to chemotherapy [11]. Reversing patients' weight loss has been shown to improve prognosis [12]. Our research indicated that effective control of tumors after four cycles of NT with FLOT significantly improved the oral feeding of patients, creating a favorable physiological condition for implementing enteral nutrition support. In the TNT group, chemotherapy cycles originally scheduled after surgery were completed before the surgery. The delayed operation strategy allowed for a period of time for nutrition support, minimizing the impact of postoperative physiological changes on chemotherapy. Patients in the TNT group experienced varying degrees of weight gain before surgery, resulting in an improved general condition for undergoing surgery. Despite a reduction in chemotherapy dose and temporary delays, all patients in the TNT group completed the established chemotherapy before the operation. In the NT group, 50% of patients were unable to complete the remaining four cycles of postoperative chemotherapy due to poor tolerance or postoperative complications. The high intensity of FLOT chemotherapy led to myelosuppression in all patients. Therefore, close monitoring of blood routine was essential to promptly address leukopenia and thrombocytopenia. It is crucial to note that the total NT strategy carries the risk of tumor progression due to delayed surgery. Hence, strict screening was implemented in our study. Patients with poorly differentiated adenocarcinoma were excluded, and only those with significant tumor shrinkage, as estimated by enhanced CT examination at the fourth chemotherapy cycle, were eligible to participate. This approach bolstered our confidence in continuing chemotherapy.

Moreover, attention should be directed towards the safety of surgery following eight cycles of NT. The European STOMACH study has affirmed that, in comparison to the direct operation group, sequential laparoscopic gastrectomy with NT does not increase postoperative complications [13]. Furthermore, the safety of TNT has been established in studies involving rectal cancer and pancreatic cancer [14, 15]. However, there is a paucity of reports in the field of GC regarding whether TNT might compromise surgical safety. As is known, the inflammatory reaction in both the tumor and its surrounding tissue can complicate the identification of the correct anatomical layer, leading to prolonged operation times and an increased risk of surgery-related complications. Prior to conducting this study, our surgical team had already performed over a thousand radical operations for GC patients, accumulating extensive surgical expertise. Moreover, a clearer surgical field due to the sufficient reduction of tumor volume allowed for a smoother surgical process. The utilization of laparoscope-assisted gastrectomy further contributed to the reduction of surgical trauma [16]. In our study, the TNT group demonstrated superiority over the NT group in terms of intraoperative blood loss and operation time, with no significant differences in overall postoperative complications. Notably, in the TNT group, one patient required transfer to the intensive care unit for treatment due to aspiration during tracheal tube removal.

In recent years, there has been a growing interest among researchers in employing total neoadjuvant therapy for patients with gastrointestinal tumors, particularly in cases of rectal cancer [17]. However, studies examining its application in GC remain relatively scarce. In the POET study [18], patients who received TNT followed by sequential chemoradiotherapy before curative surgery demonstrated a pathological complete response rate of 14%, a 3-year survival rate of 47%, and a local recurrence rate of 18.6%.Our study has also demonstrated promising results in oncology due to the utilization of a high-intensity triple-drug combination total neoadjuvant therapy. In comparison to the NT group, a significantly higher proportion of patients achieved a pathological complete regression in the TNT group (19.2% vs. 6.2%). The pathological response rate was 88% in the TNT group and 56% in the NT group. Concerning tumor stage, the TNT group exhibited a more pronounced downstaging in the T stage. Patients with pathological stage T0–T2 in the TNT group accounted for 76.9%, which was higher than the 46% in the NT group. The 3-year OS rate was 61.5% in the TNT group and 46.9% in the NT group, while the 3-year rate was 53.8% in the TNT group and 34.4% in the NT group. Although not statistically significant, the TNT group still demonstrated clinically beneficial trends in terms of long-term tumor outcomes, which deviated from the findings of the research conducted by Yang et al. [19]. The advantages in oncology might be attributed to a higher completion rate of chemotherapy and more pronounced tumor regression, reducing intraoperative tumor residues that could not be identified by the naked eye. Additionally, the patients we enrolled had more advanced tumors than those in previous studies. A longer follow-up is needed to confirm this advantage.

## 7. Conclusion

In summary, our study indicated that patients with LAGC undergoing thorough screening might derive benefits from a TNT strategy, presenting a novel treatment paradigm. In clinical practice, it is imperative to conduct a comprehensive assessment, considering factors such as patients' tolerance to chemotherapy, tumors' sensitivity to chemotherapy, and surgeons' proficiency, followed by judicious adjustments in treatment strategies, encompassing immunotherapy and radiotherapy [20, 21]. This approach is aimed at optimizing patient benefits. Nonetheless, our study has certain limitations, including a small number of cases. To validate these findings, large-scale randomized controlled studies should be undertaken.

## Figures and Tables

**Table 1:** Baseline patient characteristics in the TNT and NT groups.

**Characteristic**	**TNT (** **n** = 26**)**	**NT (** **n** = 32**)**	**p** ** value**
Age (years)			0.942
< 40	4	6	
40–60	15	18	
60–70	7	8	
Gender			0.867
Male	16	19	
Female	10	13	
NRS2002 score			0.957
2	8	9	
3	12	16	
4	6	7	
Primary site			0.897
Upper third of gastric	11	13	
Gastroesophageal junction	15	19	
Clinical T stage			0.963
cT4a	21	26	
cT4b	5	6	
Clinical N stage			0.572
cN1	3	7	
cN2	15	17	
cN3	8	8	

**Table 2:** Surgical and pathological outcomes in the TNT and NT groups.

**Variable**	**TNT (** **n** = 26**)**	**NT (** **n** = 32**)**	**p** ** value**
Operation time (min)	208 ± 21	237 ± 27	< 0.01
Estimated blood loss (mL)	73 ± 25	123 ± 56	< 0.01
Retrieved lymph nodes	36 ± 12	37 ± 14	0.812
Surgery type			0.409
Total gastrectomy	25	29	
Total gastrectomy combined with other organ resection	1	3	
R0 or R1 resection			0.681
R0	25	30	
R1	1	2	
yT stage			0.025
yT0	5	2	
yT1	6	4	
yT2	9	6	
yT3	5	12	
yT4	1	8	
yN stage			0.515
yN0	5	2	
yN1	6	9	
yN2	10	14	
yN3	5	7	
Tumor regression grade			0.039
TRG0	5	2	
TRG1	6	7	
TRG2	12	9	
TRG3	3	14	

**Table 3:** Postoperative complications in the TNT and NT groups.

	**TNT (** **n** = 26**)**	**NT (** **n** = 32**)**	**p** ** value**
Abdominal bleeding	0	1	
Abdominal infection	2	2	
Pulmonary infection	2	3	
Intestinal obstruction	0	1	
Anastomotic leakage	1	1	
Pancreatic fistula	0	1	
Lymphatic leakage	1	2	
Incision infection	1	2	
Sepsis	1	1	
Pleural effusion	3	4	
At least one of complications listed above	8	10	0.969

**Table 4:** Main adverse reactions of chemotherapy in the TNT and NT groups.

	**TNT (** **n** = 26**)**	**NT (** **n** = 32**)**	**p** ** value**
Vomiting			0.648
Grade 0	11	13	
Grade 1–2	13	14	
Grade 3–4	2	5	
Myelosuppression			0.396
Grade 1–2	16	15	
Grade 3–4	10	17	
Liver function damage			0.732
Grade 0	17	24	
Grade 1–2	8	7	
Grade 3–4	1	1	

## Data Availability

The data used to support the findings of this study are available from the corresponding author upon request.

## References

[1] Rawla P., Barsouk A. (2019). Epidemiology of Cancers of the Small Intestine: Trends, Risk Factors, and Prevention. *Przegląd Gastroenterologiczny*.

[2] Yang L., Zheng R., Wang N. (2018). Incidence and Mortality of Stomach Cancer in China, 2014. *Chinese Journal of Cancer Research*.

[3] Ajani J. A., D'Amico T. A., Almhanna K. (2016). Gastric Cancer, Version 3.2016, NCCN Clinical Practice Guidelines in Oncology. *Journal of the National Comprehensive Cancer Network*.

[4] Noh S. H., Park S. R., Yang H. K. (2014). Adjuvant Capecitabine Plus Oxaliplatin for Gastric Cancer After D2 Gastrectomy (CLASSIC): 5-Year Follow-Up of an Open-Label, Randomised Phase 3 Trial. *Lancet Oncology*.

[5] Fuentes E., Ahmad R., Hong T. S. (2016). The Impact of Neoadjuvant Therapy for Gastroesophageal Adenocarcinoma on Postoperative Morbidity and Mortality. *Journal of Surgical Oncology*.

[6] Cunningham D., Allum W. H., Stenning S. P. (2006). Perioperative Chemotherapy Versus Surgery Alone for Resectable Gastroesophageal Cancer. *New England Journal of Medicine*.

[7] Al-Batran S. E., Homann N., Pauligk C. (2019). Perioperative Chemotherapy with Fluorouracil Plus Leucovorin, Oxaliplatin, and Docetaxel Versus Fluorouracil or Capecitabine Plus Cisplatin and Epirubicin for Locally Advanced, Resectable Gastric or Gastro-Oesophageal Junction Adenocarcinoma (FLOT4): A Randomised, Phase 2/3 Trial. *Lancet*.

[8] Al-Batran S. E., Hofheinz R. D., Pauligk C. (2016). Histopathological regression After Neoadjuvant Docetaxel, Oxaliplatin, Fluorouracil, and Leucovorin Versus Epirubicin, Cisplatin, and Fluorouracil or Capecitabine in Patients With Resectable Gastric or Gastro-Oesophageal Junction Adenocarcinoma (FLOT4-AIO): Results From the Phase 2 Part of a Multicentre, Open-Label, Randomised Phase 2/3 Trial. *Lancet Oncology*.

[9] Zhang X., Liang H., Li Z. (2021). Perioperative or Postoperative Adjuvant Oxaliplatin With S-1 Versus Adjuvant Oxaliplatin With Capecitabine in Patients With Locally Advanced Gastric or Gastro-Oesophageal Junction Adenocarcinoma Undergoing D2 Gastrectomy (RESOLVE): An Open-Label, Superiority and Non-Inferiority, Phase 3 Randomised Controlled Trial. *Lancet Oncology*.

[10] Kang Y. K., Yook J. H., Park Y. K. (2021). PRODIGY: A Phase III Study of Neoadjuvant Docetaxel, Oxaliplatin, and S-1 Plus Surgery and Adjuvant S-1 Versus Surgery and Adjuvant S-1 for Resectable Advanced Gastric Cancer. *Journal of Clinical Oncology*.

[11] Soh J. Y., Cha W. C., Chang D. K. (2018). Development and Validation of a Multidisciplinary Mobile Care System for Patients With Advanced Gastrointestinal Cancer: Interventional Observation Study. *JMIR mHealth and uHealth*.

[12] Li Q. Q., Lu Z. H., Yang L. (2014). Neutrophil Count and the Inflammation-Based Glasgow Prognostic Score Predict Survival in Patients With Advanced Gastric Cancer Receiving First-Line Chemotherapy. *Asian Pacific Journal of Cancer Prevention*.

[13] van der Wielen N., Straatman J., Daams F. (2021). Open Versus Minimally Invasive Total Gastrectomy After Neoadjuvant Chemotherapy: Results of a European Randomized Trial. *Gastric Cancer*.

[14] Petrelli F., Trevisan F., Cabiddu M. (2020). Total Neoadjuvant Therapy in Rectal Cancer. *Annals of Surgery*.

[15] Truty M. J., Kendrick M. L., Nagorney D. M. (2021). Factors Predicting Response, Perioperative Outcomes, and Survival Following Total Neoadjuvant Therapy for Borderline/Locally Advanced Pancreatic Cancer. *Annals of Surgery*.

[16] Li Z., Shan F., Ying X. (2019). Assessment of Laparoscopic Distal Gastrectomy After Neoadjuvant Chemotherapy for Locally Advanced Gastric Cancer: A Randomized Clinical Trial. *JAMA Surgery*.

[17] Cercek A., Roxburgh C. S. D., Strombom P. (2018). Adoption of Total Neo-Adjuvant Therapy for Locally Advanced Rectal Cancer. *JAMA Oncology*.

[18] Stahl M., Walz M. K., Riera-Knorrenschild J. (2017). Preoperative Chemotherapy Versus Chemoradiotherapy in Locally Advanced Adenocarcinomas of the Oesophagogastric Junction (POET): Long-Term Results of a Controlled Randomised Trial. *European Journal of Cancer*.

[19] Yang J., Greally M., Strong V. E. (2023). Perioperative Versus Total Neoadjuvant Chemotherapy in Gastric Cancer. *Journal of Gastrointestinal Oncology*.

[20] Janjigian Y. Y., Shitara K., Moehler M. (2021). First-Line Nivolumab Plus Chemotherapy Versus Chemotherapy Alone for Advanced Gastric, Gastro-Oesophageal Junction, and Oesophageal Adenocarcinoma (CheckMate 649): A Randomised, Open-Label, Phase 3 Trial. *Lancet*.

[21] Stahl M., Walz M. K., Stuschke M. (2009). Phase III Comparison of Preoperative Chemotherapy Compared With Chemoradiotherapy in Patients With Locally Advanced Adenocarcinoma of the Esophagogastric Junction. *Journal of Clinical Oncology*.

